# Multifunctional on-chip directional coupler for spectral and polarimetric routing of Bloch surface wave

**DOI:** 10.1515/nanoph-2022-0397

**Published:** 2022-09-20

**Authors:** Xinrui Lei, Ruxue Wang, Li Liu, Chengjie Xu, Aimin Wu, Qiwen Zhan

**Affiliations:** School of Optical-Electrical and Computer Engineering, University of Shanghai for Science and Technology, Shanghai, 200093, P. R. China; Zhangjiang Laboratory, 100 Haike Road, Shanghai, 201204, P. R. China; Shanghai Key Lab of Modern Optical System, University of Shanghai for Science and Technology, Shanghai 200093, China; State Key Laboratory of Functional Materials for Informatics, Shanghai Institute of Microsystem and Information Technology, CAS, Shanghai, 200050, P. R. China; Center of Materials Science and Optoelectronics Engineering, University of Chinese Academy of Sciences, Beijing, 100049, P. R. China

**Keywords:** Bloch surface waves, multifunctional, nanoantenna, on-chip photonic device, spectral and polarimetric routing

## Abstract

Integration of multiple diversified functionalities into an ultracompact platform is crucial for the development of on-chip photonic devices. Recently, a promising all-dielectric two-dimensional platform based on Bloch surface waves (BSWs) sustained by dielectric multilayer has been proposed to enable various functionalities and provide novel approach to photonic devices. Here, we design and fabricate a multifunctional directional coupler to achieve both spectral and polarimetric routing by employing asymmetric nanoslits in a dielectric multilayer platform. Due to the dispersion property of BSWs, the directional coupling behavior is sensitive to wavelength and polarization. We demonstrate numerically and experimentally the wavelength selective directional coupling of TE BSW mode with an intensity ratio of the BSW excitation in opposite directions reaching 10 dB. Polarization selective directional coupling is also achieved at specific operating wavelength due to different response to a nanoantenna for TE and TM BSWs. The proposed two-dimensional photonic device opens new pathway for a wide range of practical applications such as molecular sensing, imaging with different polarization, and spectral requirements.

## 1 Introduction

Integration and miniaturization of on-chip photonic devices are of great importance with the demand of increased speed and capability in information acquisition, processing, and storage. Among the numerous approaches to enable ultracompact devices, the employment of two-dimensional surface electromagnetic waves like surface plasmon polaritons (SPPs) has been intriguing and widely investigated, for the ability to overcome diffraction limit and confine light at subwavelength scale [1, 2]. Many typical 2D plasmonic devices were proposed such as light emitters [3, 4], photodetectors [5], modulators [6, 7], biosensors [8, 9], lenses [10, 11], dichroic splitters [12–18], and beam splitters [19–22]. Since great achievements have been made to realize customized single functionality in plasmonic devices, more and more efforts were devoted to enabling multiple diversified functionalities into an ultracompact platform. To this end, plasmonic metasurfaces composed of planar arrays of resonant elements were proposed to integrate different channels and deal with concurrent tasks [23–29]. However, metasurface-based approaches may suffer from intrinsic crosstalk between different subarrays and sophisticated fabrication technology due to the ultra-fine unit cell especially in the visible range. On the other hand, the use of metal is inevitably accompanied by Ohmic losses and makes it difficult to be compatible with the commercial CMOS platform.

As an alternative approach, all-dielectric two-dimensional photonic platform based on Bloch surface waves (BSWs) was proposed and developed. BSWs are surface electromagnetic waves excited at the interface between a truncated periodic dielectric multilayer and a dielectric medium [30]. As a dielectric counterpart to SPPs, BSWs can also enable optical near-field confinement and enhancement, while exhibiting longer propagation length and higher resonance quality factor [31–36]. BSWs can be either transverse electric (TE) and transverse magnetic (TM) polarization [37, 38] with the dispersion relations tuned by altering the multilayer parameters, giving rise to an ultrawide excitation frequencies range [39, 40]. Such appealing characteristics allow for many BSW-based 2D photonic devices [41–44], enabling various functionalities including sensing [45–47], subwavelength focusing [48–51], surface-enhanced Raman scattering [52, 53], directional coupling [54, 55], and lasing [56]. Moreover, the dispersion property and polarization diversity of BSWs may provide novel degree of freedom for integrating multiple functionalities by compact nanostructures.

In this paper, we propose an ultracompact directional coupler based on the BSW platform, which can realize simultaneous spectral and polarimetric routing. The directional excitation of BSW is achieved by employing a pair of asymmetric nanoslits with each nanoslit acting as a nanoantenna to couple free space light to surface mode. We analyze the excitation of BSWs on the nanoslits in the dielectric multilayer with effective refractive index approach, and reveal that the directional coupling behavior is sensitive to both wavelength and polarization, giving rise to spectral and polarimetric routing of BSWs. By carefully engineering the geometry of nanoslits, we demonstrate both numerically and experimentally the wavelength selective directional coupling of TE BSW mode with directivity more than 10 dB for the operating wavelength. Meanwhile, polarization selective directional coupling is achieved due to the different response to a nanoantenna for TE and TM BSWs, with a directivity reaching up to 8.5 dB. Our approach paves the way for the development of multiplexing and multifunctional on-chip photonic devices, which can be potentially applied in optical communication, sensing and photonic circuits.

## 2 Results and discussion

**Figure 1:**
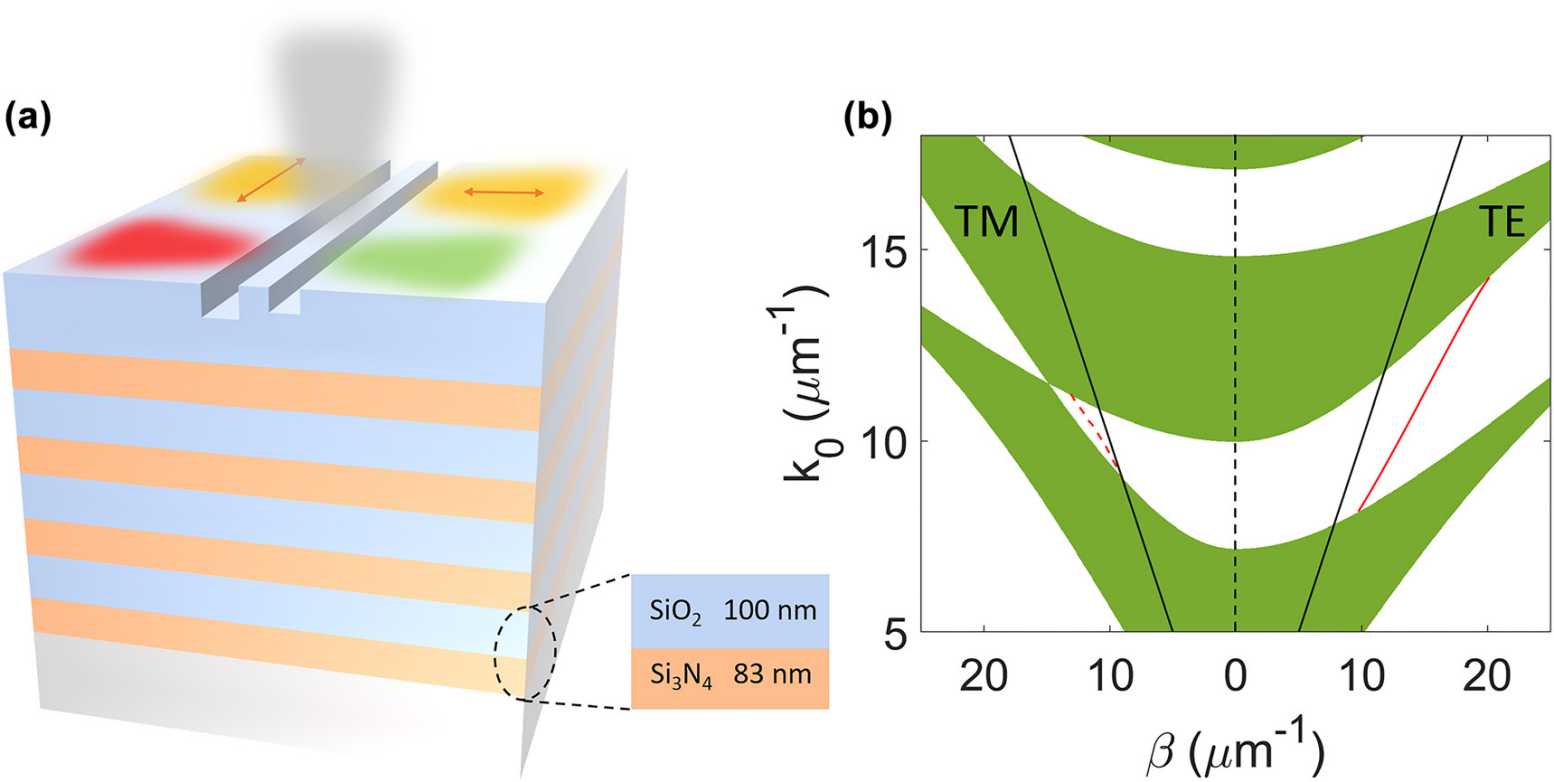
Schematics of the multifunctional on-chip directional coupler. (a) The proposed configuration consists of a pair of asymmetric nanoslits on the top layer of a dielectric multilayer, which launches BSWs preferentially to the left or right depending on the wavelength or polarization of the incident free-space beam. (b) Simulated photonic band structures of the considered dielectric multilayer for TE (right) and TM (left) polarizations. The white regions correspond to the photonic forbidden bands, where light cannot propagate through the multilayer, and the green regions represents the allowed bands. The red solid and dashed lines denote the dispersion curve for the TE and TM BSWs respectively. The black solid lines denote the light line in vacuum.

A schematic view of the proposed on-chip directional coupler is shown in Figure 1(a). By breaking the mirror symmetry of nanoantennas, normally incident free-space light is directionally coupled to BSWs depending on the wavelength or polarization. The dielectric multilayer that sustains both TE and TM polarized BSWs consists of 8 alternating layers of SiO_2_ (low refractive index, *n* = 1.46) and Si_3_N_4_ (high refractive index, *n* = 2.7 at a wavelength of 640 nm but dispersive. See Figure S1 for measured refractive index) on a cover glass (the number of the layer pairs is less than commonly used in order to increase the leakage loss, ensuring both TE and TM polarized BSW can be detected by the leakage radiation microscopy system, while the wavevector of BSWs is approaching that for large number of the layer pairs. See Figure S2 for details). The thicknesses of the alternating SiO_2_ and Si_3_N_4_ layers are 100 and 83 nm, respectively, and the thickness of the top SiO_2_ is 360 nm (Figure 1(a)). The photonic band structures of the dielectric multilayer for both TE and TM modes are shown in Figure 1(b). The white zones denote the photonic forbidden band of the dielectric multilayer, where the Bloch wave numbers are imaginary and electromagnetic fields decay exponentially away from the interface of top layer, hence surface mode can exist, while the green zones denote the photonic allowed bands where light can propagate inside the multilayer. The dispersion relations of BSWs are calculated in Figure 1(b) via transfer matrix method (red solid and dashed lines), which are located below the vacuum light lines (black lines). It can be obtained from the dispersion curves that the TM-BSWs are sustained in a narrower wavelength range (from 560 to 690 nm) than that of TE-BSWs (from 440 to 770 nm), which is attributed to the smaller band width for TM mode due to the Brewster effect.

**Figure 2:**
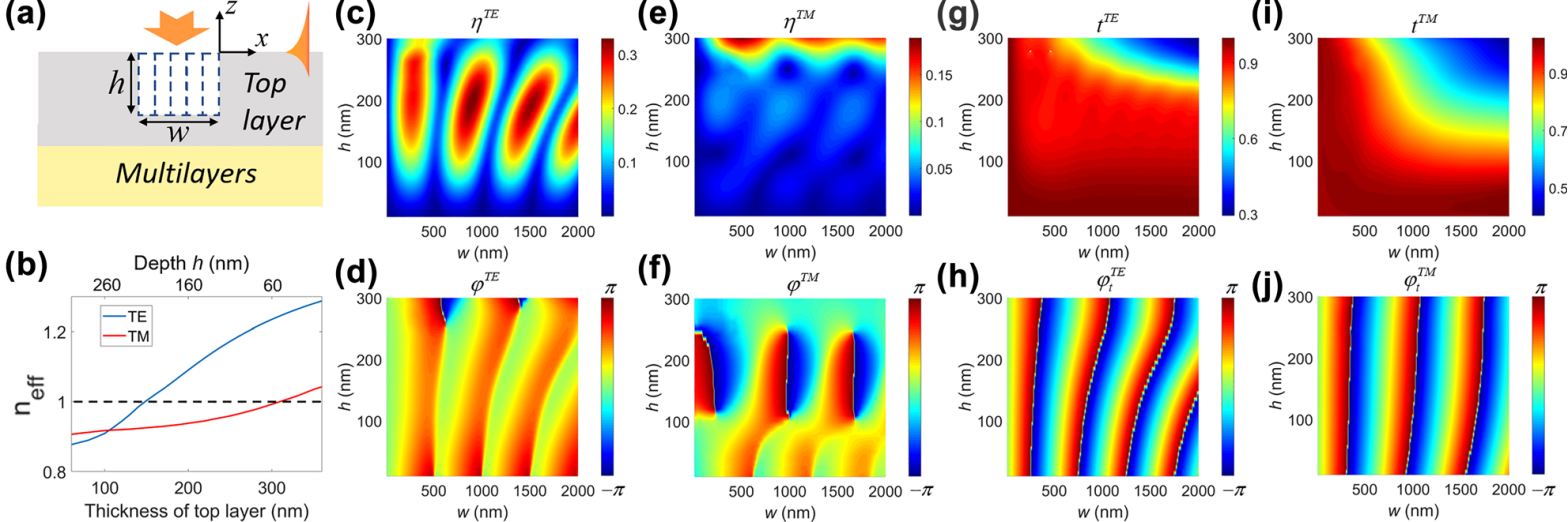
The excitation process of BSWs by a single nanoslit. (a) Schematics of BSW generation by nanoslit under plane wave illumination, which can be regarded as a superposition of narrow slit launchers. Each launcher is represented as the dashed rectangles. (b) Effective refractive index dependence on the thickness of the top layer for both TE and TM modes for a wavelength of 640 nm. The black dashed line represents *n*_eff_ = 1, below which corresponds to the radiation mode. It is worth noting that only TM_1_ BSW mode is considered here. (c–f) Dependence of coupling efficiency *η* and phase *φ* of BSWs on the slit width and depth under (c–d) TE and (e–f) TM polarized plane wave illumination. The amplitude of plane wave is set as 1. (g–j) Transmittance *t* and transmission phase *φ*_*t*_ of (c–d) TE and (e–f) TM polarized BSWs when passing through a slit for varying width and depth. The wavelength in vacuum is 640 nm in (c–j). The right edge of slit is fixed at *x* = 0 with increasing width in (c–j).

A nanoslit etched in the multilayer structure is the basic element of on-chip coupler, which acts as a nanoantenna and couples an incident free-space beam to BSWs propagating on the surface. The excitation process of BSWs by a rectangular nanoslit (assume the slit extends infinitely along *y* axis) under plane wave illumination can be considered as a superposition of narrow slit launchers, as shown in Figure 2(a). BSWs excited from each launcher will propagate inside the slit and then along the surface of multilayer. The *y* component of electromagnetic field at position (*x*, *y*, 0) can be expressed as (*x >* 0 and far from slit edge)

(1) $$\begin{array}{ll} \Psi_{y}\left(x\right) & =\alpha {\text{e}}^{\text{i}k_{\text{BSW}}x}\overset{0}{\underset{-w}{\int }}{\text{e}}^{\gamma \zeta /w}{\text{e}}^{-ik_{in}\zeta }d\zeta \\ & =\alpha {\text{e}}^{\text{i}k_{\text{BSW}}x}\frac{{\text{e}}^{-\gamma }{\text{e}}^{\text{i}k_{in}w}-1}{ik_{in}-\gamma /w}, \end{array}$$

where Ψ_*y*_ denotes electric field for TE mode and magnetic field for TM mode, *α* is a constant, *w* is the width of slit, *k*_BSW_ is the wavevector of BSW, *k*_in_ is the wavevector inside the slit, *γ* is a decay coefficient. Photon-BSW coupling can be quantified from Equation (1) by the coupling efficiency *η* ∼ |Ψ_*y*_| which denotes the field amplitude ratio between the excited BSW and incident light, as well as the coupling phase *φ* ∼ Arg[Ψ_*y*_] representing the initial phase of the excited BSW. From Equation (1), the coupling efficiency and phase will oscillate on slit width with period of 2*π*/*k*_in_. For a truncated periodic multilayer, the thickness of top layer determines the location of the dispersion line in the band gap and effective refractive index of mode. Figure 2(b) demonstrates the effective indices dependence on the thickness of top layer for both TE and TM modes at a wavelength of 640 nm, which decrease with the decreasing thickness. Numerical simulations were also performed by finite difference time domain (FDTD) method to understand the excitation process of BSWs. Under normally incident plane wave illumination, BSW coupling efficiency *η* and phase *φ* variations with the slit width and depth are shown in Figure 2(c–f) for a wavelength of 640 nm, which coincides well with theoretical predictions (see Methods for details of the simulations). It is worth noting that the coupling efficiency for TM BSWs increases with slit depth in the considered range, making it not visible as slit depth is shallow. The segmented diagram of coupling efficiency varying on *w* and *h* for each short range of slit depth is shown in Figure S3. In addition to the launching of BSWs, a nanoslit can reflect and transmit incident BSWs. As an incident BSW is propagating along the positive *x* axis and crossing a slit in Figure 2(a), simulated complex transmission coefficient variation on slit geometry is shown in Figure 2(g–j) for a wavelength of 640 nm, which can be approximated as $\tilde{t}\approx t{\text{e}}^{\text{i}k_{in}w}$ with *t* denoting the transmittance. It can be obtained from Figure 2(g) and (i) that the transmittance *t* will decrease with increasing slit size.

**Figure 3:**
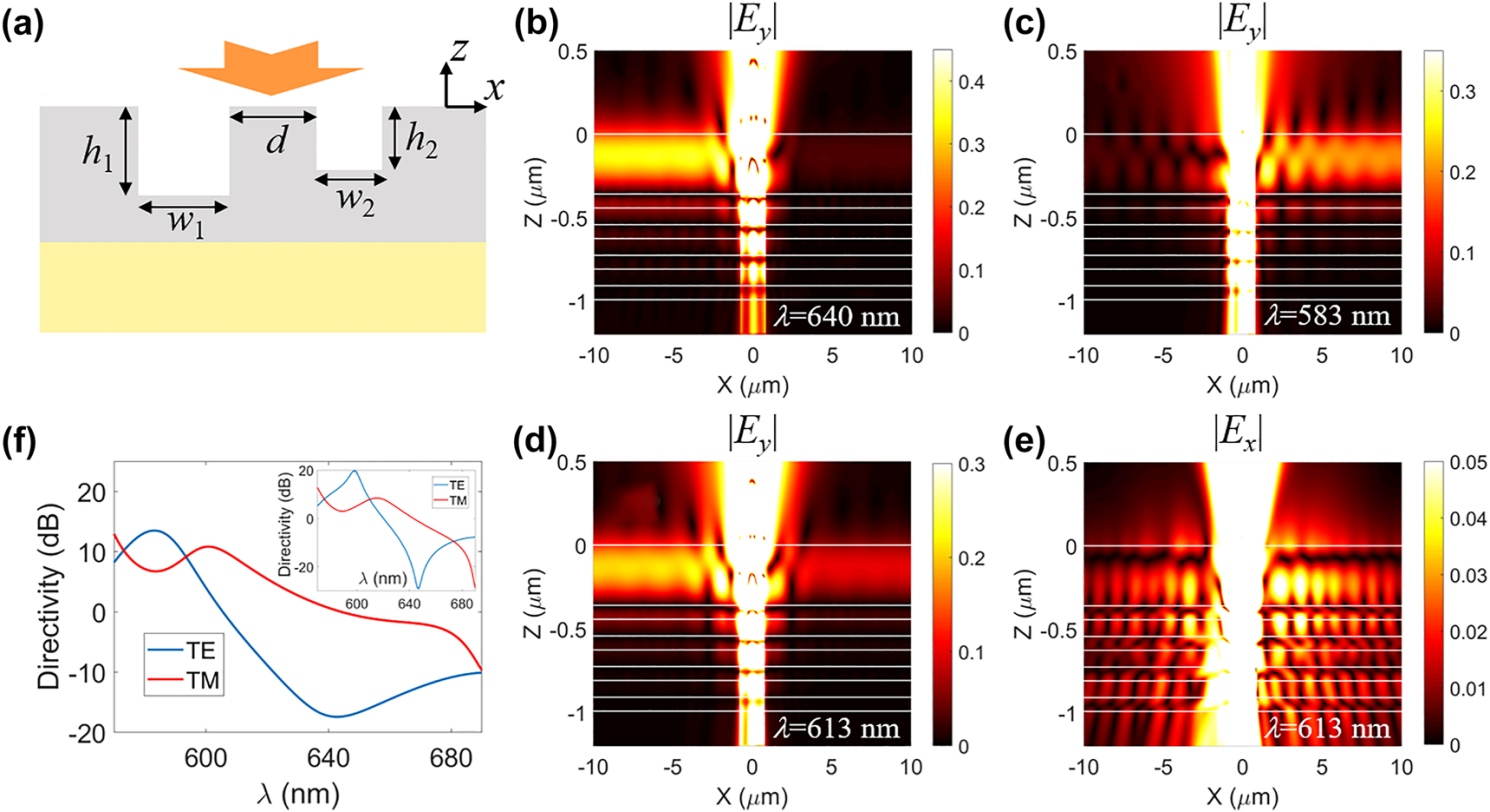
Directional excitation of BSWs by asymmetric nanoslit pair. (a) Schematic diagram of the configuration. (b–c) Simulated near-field distribution of the electric field *E*_*y*_ in *x–z* plane across the multilayer under the illumination with a normally incident TE polarized plane wave for a wavelength of (b) 640 nm and (c) 583 nm. (d–e) Simulated near-field distribution of the electric field (d) *E*_*y*_ and (e) *E*_*x*_ in *x–z* plane under the illumination with a normally incident (d) TE and (e) TM polarized plane wave for a wavelength 613 nm. The directivities for the two operation wavelengths in (b) and (c) as well as two orthogonal polarizations in (d) and (e), are opposite, exhibiting both spectral and polarimetric routing behavior. The white solid lines in (b–e) denote the interfaces of each layer. (f) Wavelength dependence of the directivity for both TE (blue solid line) and TM (red solid line) polarized BSWs. The width and depth of the slits are *w*_1_ = 540 nm, *h*_1_ = 250 nm, *w*_2_ = 480 nm, *h*_2_ = 130 nm, and the length of SiO_2_ ridge between the slits is *d* = 260 nm. The amplitude of incident plane wave is set as 1 in (b–f). Inset of (f) depicts the wavelength dependence of the directivity with *d* = 320 nm while maintaining the width and depth of nanoslits, demonstrating a red shift of spectral band.

Directional coupling of BSWs can be achieved by breaking the mirror symmetry of nanoslits, since the scattering behavior of BSWs strongly relies on the slit geometry. We consider a pair of nanoslits with different width and depth illuminated by an incident plane wave, as shown in Figure 3(a). The BSW field propagating along the ±*x* directions can be expressed as

(2a) $$\Psi_{l}=\eta_{1}+\eta_{2}t_{1}{\text{e}}^{\text{i}\left(k_{\text{BSW}}d+k_{in,1}w_{1}+\varphi_{2}-\varphi_{1}\right)}=\eta_{1}+\eta_{2}t_{1}{\text{e}}^{\text{i}\Delta \varphi_{l}}$$

(2b) $$\Psi_{r}=\eta_{2}+\eta_{1}t_{2}{\text{e}}^{\text{i}\left(k_{\text{BSW}}d+k_{in,2}w_{2}+\varphi_{1}-\varphi_{2}\right)}=\eta_{2}+\eta_{1}t_{2}{\text{e}}^{\text{i}\Delta \varphi_{r}}$$

where *d* is the length of SiO_2_ ridge between the slits, *η*_*i*_ and *φ*_*i*_ (*i* = 1, 2) are coupling efficiency and phase of each slit, *w*_*i*_ and *h*_*i*_ are width and depth of each slit, *t*_*i*_ is the transmittance for the BSW crossing slit *i*. To realize unidirectional excitation, BSWs propagating in one direction should be suppressed by destructive interference, whereas constructive interference should occur on the other side. As the slit geometry is at subwavelength scale, the transmittance of BSW can be approximated as 1, and largest extinction ratio can be obtained with *η*_1_ ≈ *η*_2_ and the relative phase terms in Equation (2) being 0 and *π* respectively. Different with the directional coupling of SPPs or waveguides [57–66], the dispersion relation of BSW can be flexibly tuned by thickness of top layer and polarization with the effective refractive index sensitive to wavelength, giving rise to additional degree of freedom to manipulate the directional coupling behavior of BSWs and integrate more functionalities in an ultracompact platform. One the one hand, the extinction ratio is highly sensitive to wavelength due to the rapid changes of *k*_BSW_ with wavelength, giving rise to spectral routing of BSWs. From Equation (1), although *η* and *φ* for individual slit vary with wavelength, coupling phase difference between the nanoslits Δ*φ* = *φ*_2_ − *φ*_1_ barely changes as the slits take close width. Consequently, Δ*φ*_*r*_ and Δ*φ*_*l*_ for interference on both sides will increase with decreasing wavelength, which results in a reversal of directionality at two operating wavelengths. On the other hand, TE and TM BSWs take different dispersion relations, leading to different interference patterns for each wavelength. This enables both spectral and polarimetric routing by carefully engineering slit parameters. We select the widths and depths of the slits as *w*_1_ = 540 nm, *h*_1_ = 250 nm and *w*_2_ = 480 nm, *h*_2_ = 130 nm, while the length of SiO_2_ ridge between the slits *d* = 260 nm. As shown in Figure 3(b–c), simulated near-field distribution of *E*_*y*_ under normal plane-wave illumination polarized along *y*-axis at 640 nm exhibits highly directionality with BSW propagating to the left (−*x* direction), while at a wavelength of 583 nm the BSW is coupled to the right (+*x* direction), demonstrating a spectral selective directional coupling. Meanwhile, the near-field distributions of *E*_*y*_ and *E*_*x*_ under normal plane-wave illumination polarized along *y*- and *x*-axis at 613 nm are shown in Figure 3(d–e), where TE and TM polarized BSWs are preferentially generated towards the left and right respectively. The interference of opposite propagating waves inside the top layer reduces the visibility of directional coupling in Figure 3(e). As an alternate demonstration, the near-field distribution of *E*_*z*_ is shown in Figure S4. To quantify the directional coupling performance, the directivity is defined as the ratio of the intensities of BSWs propagating in opposite directions

(3) $$D=10\log_{10}\left(E_{r}^{2}/E_{l}^{2}\right)$$

where *E*_*r*_ and *E*_*l*_ denote mean electric field along positive and negative *x* directions at *z* = 0 plane, respectively. Simulated directivity variations on wavelength for both TE and TM BSWs are shown in Figure 3(f), demonstrating a high spectral sensitivity of directivity which is attributed to the dispersion property of BSWs. To maximize the directivity, the electromagnetic field in one direction should be suppressed to zero, yielding *η*_1_ = *η*_2_ and Δ*φ*_*r*_ = *π* (or Δ*φ*_*l*_ = *π*) simultaneously. By optimizing the slit parameters, it is facile to improve directivity at specific wavelength. However, the directivity at other operating wavelengths may decline since the coupling efficiency *η* for the two nanoslits take different variation tendency on wavelength. To stabilize the performance of spectral and polarimetric routing, the directivity in this study is compromised to operate at multiple wavelengths. From Equation (2), the spectral band can be red or blue shifted by increasing or decreasing the length of SiO_2_ ridge *d*. The inset of Figure 3(f) shows the directivity variations on wavelength for *d* = 320 nm, demonstrating a red shift of spectral band for both spectral (operating at 600 and 647 nm) and polarimetric routing (operating at 628 nm). By varying the parameters of multilayers or nanoslits, the spectral band can also be tuned in a more flexible way. Multiple spectral bands can be integrated accordingly by arranging different nanoslit-pairs on the multilayer platform.

**Figure 4:**
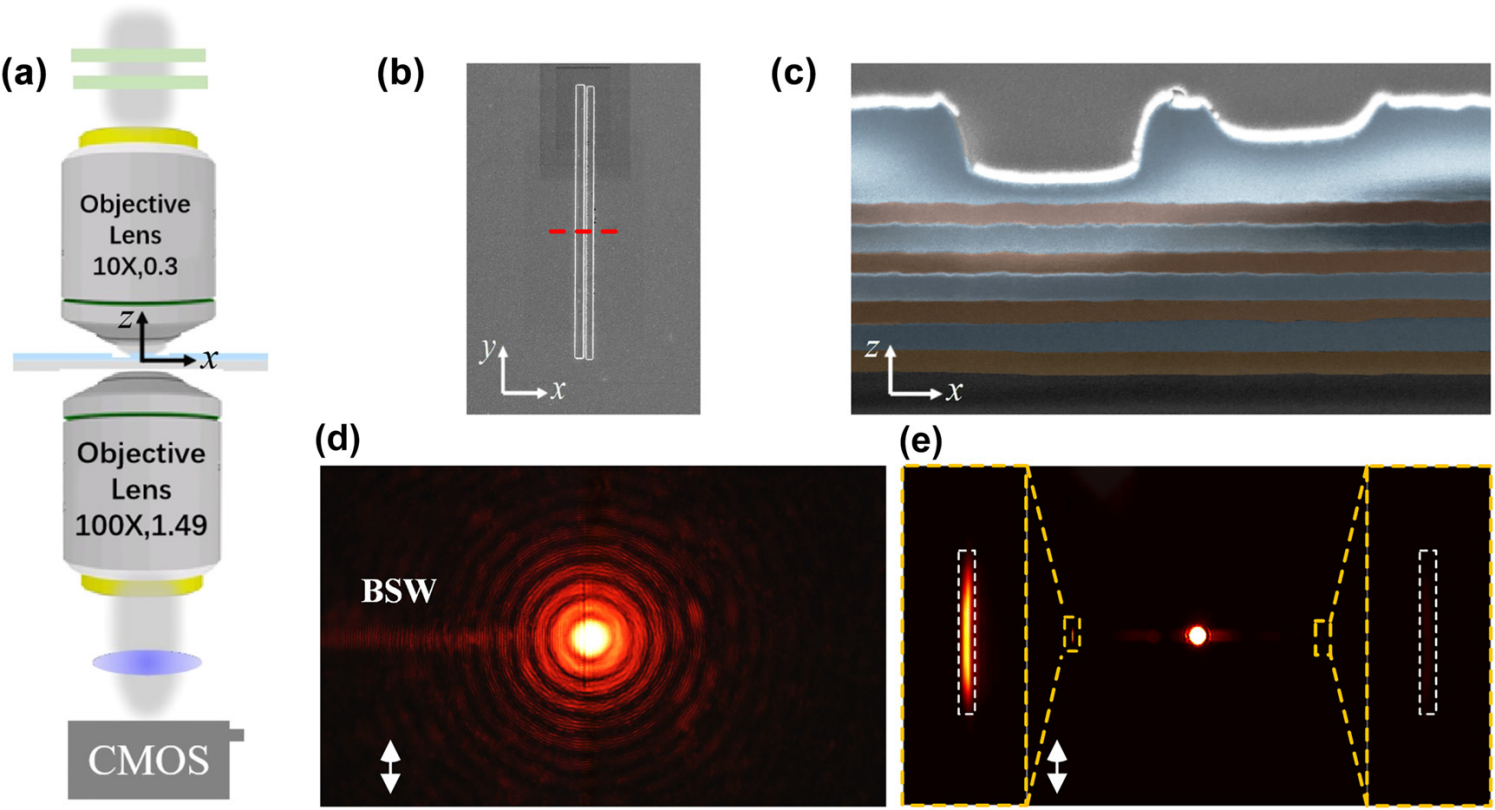
Experimental demonstration of the directional coupling of BSWs. (a) Schematic diagram of the experimental setup. (b) Top view of the SEM image of asymmetric slits. (c) Cross-sectional SEM image of the slits and the dielectric multilayer along the red dashed line in (b). (d–e) FFP and BFP images of the excited BSW under the illumination of TE polarized incident beam at a wavelength of 640 nm. The yellow dashed rectangles on the two sides in (e) show the enlarged views of BSW signals and the white dashed rectangles represent the accumulated area to calculate the intensity of BSWs. The white double arrows indicate the polarization of the incident beam in (d–e).

We experimentally demonstrated the directional coupling of BSW with the leakage radiation microscopy (LRM), which is schematically shown in Figure 4(a). A collimated laser beam (NKT supercontinuum laser) was focused onto the sample with an objective (NA = 0.3, 10x), leading to the directional excitation of BSW that partially radiates (leaks) into the substrate. A polarizer and half-wave plate were inserted into the incident beam to alter the polarization without changing the intensity. The propagation direction of the incident beam is along *z*-axis and BSWs is propagating along *x*-axis perpendicular to the long axis of the nanoslits. The propagation of BSWs was collected by leakage radiation with an oil-immersion objective (NA = 1.49, 100x). The intensity of leakage radiation is proportional to the BSW near-field intensity. Both front focal plane (FFP) and back focal plane (BFP) images were collected by switching the focal length of lens. The dielectric multilayer was fabricated via plasma-enhanced chemical vapor deposition and asymmetric slits were fabricated on the dielectric multilayer by focus ion beam (see Methods for the details). The top-view and cross-sectional scanning electron microscope (SEM) images of the sample were shown in Figure 4(b–c), in consistent with the theoretical design, except for accumulated surface roughness. Figure 4(d) and (e) are the FFP and BFP of the TE-BSWs under the illumination of *y*-polarized beam at a wavelength of 640 nm, which confirms the directional excitation of BSWs as predicted in Figure 3(b). From the FFP image in Figure 4(d), the propagation length of the BSWs can be estimated as 38.7 μm (see Figure S5 for details). The energy coupling efficiency of the coupler can be quantified by averaging the intensity of excited BSW signals in Figure 4(d) as *C* = *I*_BSW_/*I*_in_ *σ* = 3.6% (slightly lower than simulation value of 4.5%), where *P*_BSW_ and *P*_in_ are averaging power for BSWs and incident light, respectively, *σ* is a correction term for the exponential damping of BSWs in propagation. The BSW signal in the BFP image is enlarged indicated by the yellow dotted rectangle in Figure 4(e). The directivity can be calculated from BFP as *D* = 10 log_10_(*I*_*r*_/*I*_*l*_) = −12.22 dB, where *I*_*l*_ and *I*_*r*_ are the total intensities of the BSWs in the opposite directions in *k* space with |*k*_*x*_ − *k*_BSW_| < 0.008*k*_0_ and |*k*_*y*_| < 0.08*k*_0_ (the white dashed rectangles in Figure 4(e)).

**Figure 5:**
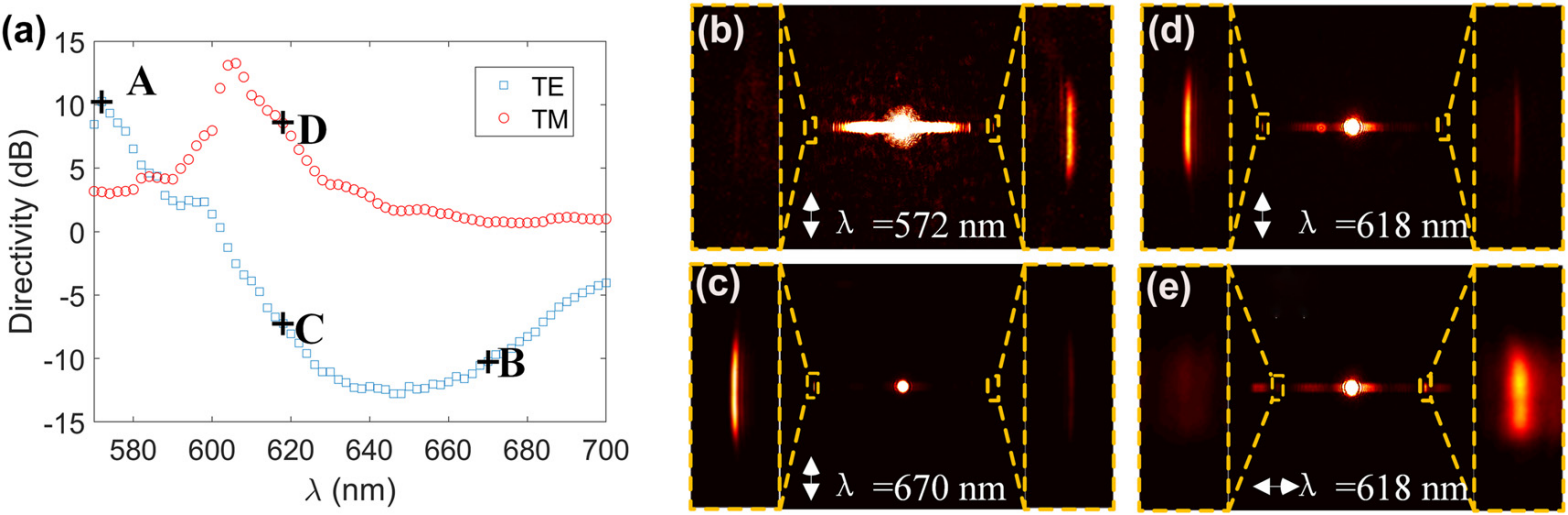
Experimental demonstration of spectral and polarimetric routing behavior of the BSWs. (a) Directivity variation on incident wavelength for TE (blue rectangles) and TM (red circles) BSWs. (b–e) The BFP images for different wavelength and polarization marked as A, B, C, D in (a), respectively, exhibiting opposite directivity for different wavelength in (b–c) and orthogonal polarization in (d–e). The BFP image in (b) is slightly overexposed to increase the visibility of BSW signal. The yellow dashed rectangles on the two sides in (b–e) show the enlarged views of BSW signals. The white double arrows indicate the polarization of the incident beam in (b–e).

To demonstrate spectral and polarimetric routing behavior, incident beam polarized along both *x* and *y*-axis (corresponding to TM and TE polarization) with wavelength ranging from 570 to 700 nm stepped by 2 nm was employed as incident light. The BFP is used to characterize the propagation of BSWs instead of the FFP due to the weak scattering field in the FFP. The directivity variations on wavelength for both TE and TM BSWs are shown in Figure 5(a) which coincide well with theoretical prediction (The transverse scale of accumulation area to calculate the intensity of TM BSWs is slightly tuned to |*k*_*x*_ − *k*_*BSW*_| < 0.012*k*_0_ due to the large propagation loss, which can be greatly reduced by increasing the number of alternating layer [38] while maintaining the directivity of system. In this work, only four pairs of alternating layers were used in order to detect both TE and TM BSW signals in LRM). Broadband spectral routing behavior is observed for TE BSWs with *D* = 10.26 dB at 572 nm and *D* = −10.23 dB at 670 nm, with the BFP images shown in Figure 5(b–c) respectively. In addition, broadband unidirectional excitation of TE BSWs is achieved from 626 to 670 nm with the directivity of less than −10 dB. Polarimetric routing is obtained at wavelength of 618 nm. From the BFP images in Figure 5(d–e), TE BSW propagates preferentially toward left with *D* = −7.25 dB while TM BSW propagates oppositely with *D* = 8.54 dB, demonstrating low crosstalk for orthogonal polarization. Due to the beam profile of Gaussian beam, the waist and position of the incident light will slightly affect the absolute directivity for each wavelength, while maintaining the spectral and polarimetric routing patterns since there is no phase modulation (see Figures S6 and S7 for details). The roughness and thickness variation over the sample area will also make influence on the directional coupling behavior, since it will introduce a tilt of the incident beam with respect to the nanostructure, which results in additional relative phase between the nanoslits. Different tilt angle would increase or suppress the directional coupling for TE or TM BSWs, which is another pathway to manipulate the excitation of BSWs operating for specific wavelength and polarization (see Figure S8 for details).

## 3 Conclusion and outlook

In summary, we have demonstrated both theoretically and experimentally a multifunctional on-chip directional coupler for spectral and polarimetric routing. The ultracompact device consists of a pair of asymmetric nanoslits perforated on a dielectric multilayer, which can directionally couple free space light to BSWs. The dispersion property of BSWs results in a large directivity and provides high sensitivity to illumination wavelength, enabling two wavelength-encoded TE BSW signals coupled in opposite directions with directivity more than 10 dB. Meanwhile, different responses to a nanoantenna for TE and TM BSWs give rise to polarization selective directional coupling with a directivity reaching up to 8.5 dB. In addition, the spectral band can be tuned by adjusting the parameters of nanoslits and multilayer, and multiple spectral bands can be integrated accordingly by arranging different nanoslit-pairs on the multilayer platform. The proposed platform can also be applied for metrology by introducing relative displacement between the incident Gaussian beam and structure which will lead to a rapid change of directivity. Owing to the compactness and integration compatibility, the proposed BSW-based directional coupler operating at wide spectral range promise high performance, low crosstalk, and polarization/wavelength-controlled functionalities for more advanced applications related to integrated photonics circuits.

## 4 Methods

### 4.1 Numerical simulations

The electromagnetic simulations are performed using two-dimensional finite difference time domain (FDTD) method. The boundary conditions in *x* and *z* directions are set as PML absorbing boundary conditions. To evaluate the coupling efficiency and phase, the nanoslit etched on the SiO_2_ layer is illuminated at normal incidence by a linearly polarized plane wave above the sample. A point monitor is placed at the SiO_2_/air interface and 10 μm away from the right edge of the slit to measure coupling phase, while the coupling efficiency is calculated by integrating the intensity of BSW signals along a line monitor ranging from 5 to 15 μm away from the edge of the slit. To calculate the complex transmission coefficient of BSW when passing through a nanoslit, the electromagnetic fields at the point monitor with and without the nanoslit are both measured as Ψ_1_ and Ψ_0_, and the transmission coefficient can be obtained as $\tilde{t}=\Psi_{1}/\Psi_{0}{\text{e}}^{\text{i}k_{\text{BSW}}w}$ . A non-uniform mesh is used throughout the whole simulation domain and the minimum mesh size is 0.5 nm.

### 4.2 Sample fabrication and characterization

The dielectric multilayers were fabricated by plasma-enhanced chemical vapor deposition (PECVD). Silicon dioxide (SiO_2_) and silicon nitride (Si_3_N_4_) were deposited on a standard microscope cover glass (0.17 mm thickness) at a vacuum <0.1 mtorr and temperature of 300 °C. The low refractive index layer was SiO_2_ (*n* = 1.46), and high refractive index was Si_3_N_4_ (*n* = 2.7 at a wavelength of 640 nm). There are 8 layers in total with the thicknesses of alternating SiO_2_ and Si_3_N_4_ 100 and 83 nm, respectively. The thickness of the top SiO_2_ layer was 360 nm. The asymmetric nanoslits were fabricated using a Focused ion beam (FIB). The dielectric multilayer was then coated with a gold film of 20 nm thickness by magnetron sputtering (Sputter–Lesker Lab 18) to conduct electricity for observation. After the etch, the gold film was removed with aqua regia. The top-view and cross-section of the sample were characterized by SEM, and the cross section is cut by the FIB.

### 4.3 Leakage radiation microscopy

We employed a leakage radiation microscope (LRM) for BSW studies as well as spectral and polarimetric routing demonstration. A collimated laser beam (NKT supercontinuum laser) was focused by a microscope objective (×10, numerical aperture NA = 0.3) with different wavelengths. A linear polarizer and half-wave plate were inserted into the incident beam to change the polarization without altering the intensity and the BFPs were captured for the demonstration of polarimetric routing. In order to demonstrate the functionality of spectral routing, we also captured the BFPs by changing the incident wavelength with a step of 2 nm across 570–700 nm. A second microscope objective (oil-immersion, ×100, NA = 1.49) below the substrate was used to collect both the transmitted beam and excited BSW. Two lenses with different focal length were used for the FFP imaging (Retiga camera, Qimaging, America) and BFP imaging (Neo-sCMOS camera, Andor, UK).

## Supplementary Material

Supplementary Material Details
