# Supplementary material for: Multifunctional on-chip directional coupler for spectral and polarimetric routing of Bloch surface wave
Source: Nanophotonics. 2022 Sep 20;11(21):4627–36. doi: 10.1515/nanoph-2022-0397 (PMC11501675; doi:10.1515/nanoph-2022-0397)
Supplement: Supplementary file 1 — Supplementary Material Details [file j_nanoph-2022-0397_suppl.docx]

Supplementary Information for

**Multifunctional on-chip directional coupler for spectral and polarimetric routing of** **Bloch surface wave**

Xinrui Lei^1, 2, 3, #^, Ruxue Wang^4, 5, #^, Li Liu^4, 5^, Chengjie Xu^4, 5^, Aimin Wu^4, 5, *^, Qiwen Zhan^1, 2, 3, *^

^1^School of Optical-Electrical and Computer Engineering, University of Shanghai for Science and Technology, Shanghai, 200093, P. R. China

^2^Zhangjiang Laboratory, 100 Haike Road, Shanghai, 201204, P. R. China

^3^Shanghai Key Lab of Modern Optical System, University of Shanghai for Science and Technology, Shanghai 200093, China

^4^State Key Laboratory of Functional Materials for Informatics, Shanghai Institute of Microsystem and Information Technology, CAS, Shanghai, 200050, P. R. China

^5^Center of Materials Science and Optoelectronics Engineering, University of Chinese Academy of Sciences, Beijing, 100049, P. R. China


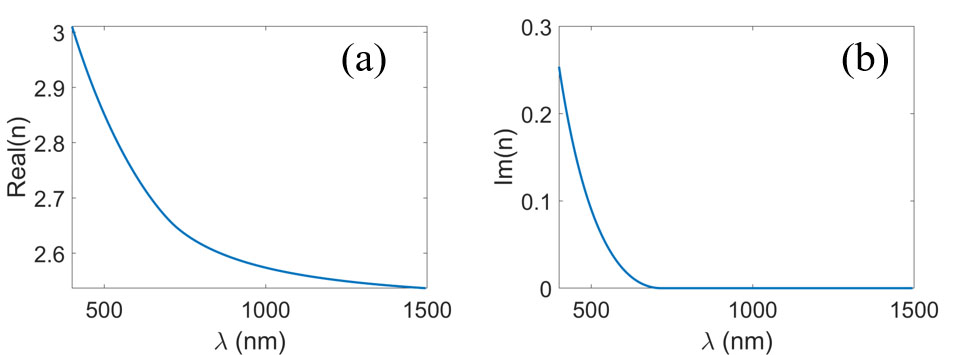


**Supplementary Figure S1.** Measured (a) real part and (b) imaginary part of refractive index dependence of on wavelength for Si_3_N_4_.


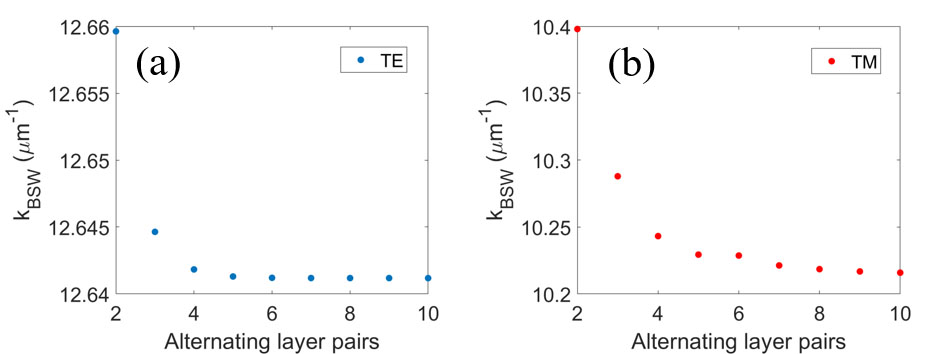


**Supplementary Figure S2.** Wavevector dependences on the number of alternating layer pairs for (a) TE and (b) TM BSWs at a wavelength of 640 nm calculated via transfer matrix method.


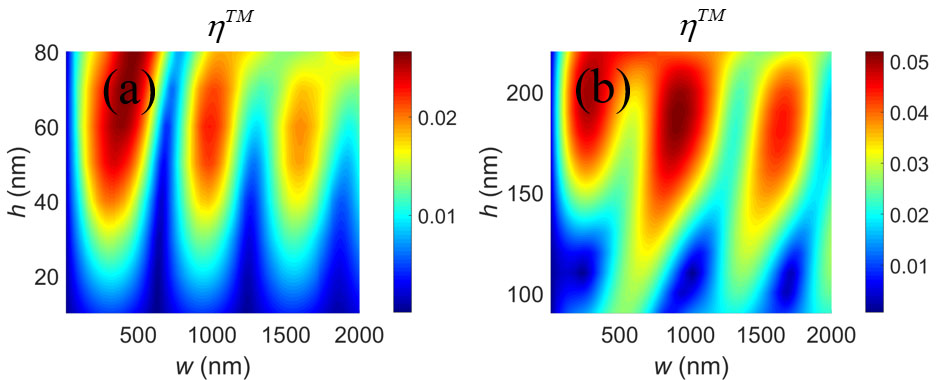


**Supplementary Figure S3.** Dependence of coupling efficiency *η* for TM BSWs on the slit width and depth at a wavelength of 640 nm for (a) *h* < 80 nm (b) 80 nm < *h* < 220 nm.


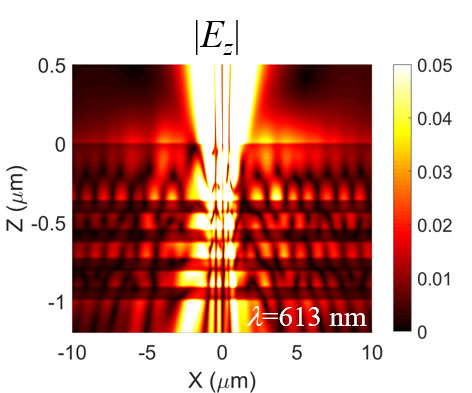


**Supplementary Figure S4.** Simulated near-field distribution of the electric field *E_z_* under the illumination with a normally incident TM polarized plane wave for a wavelength 613 nm.


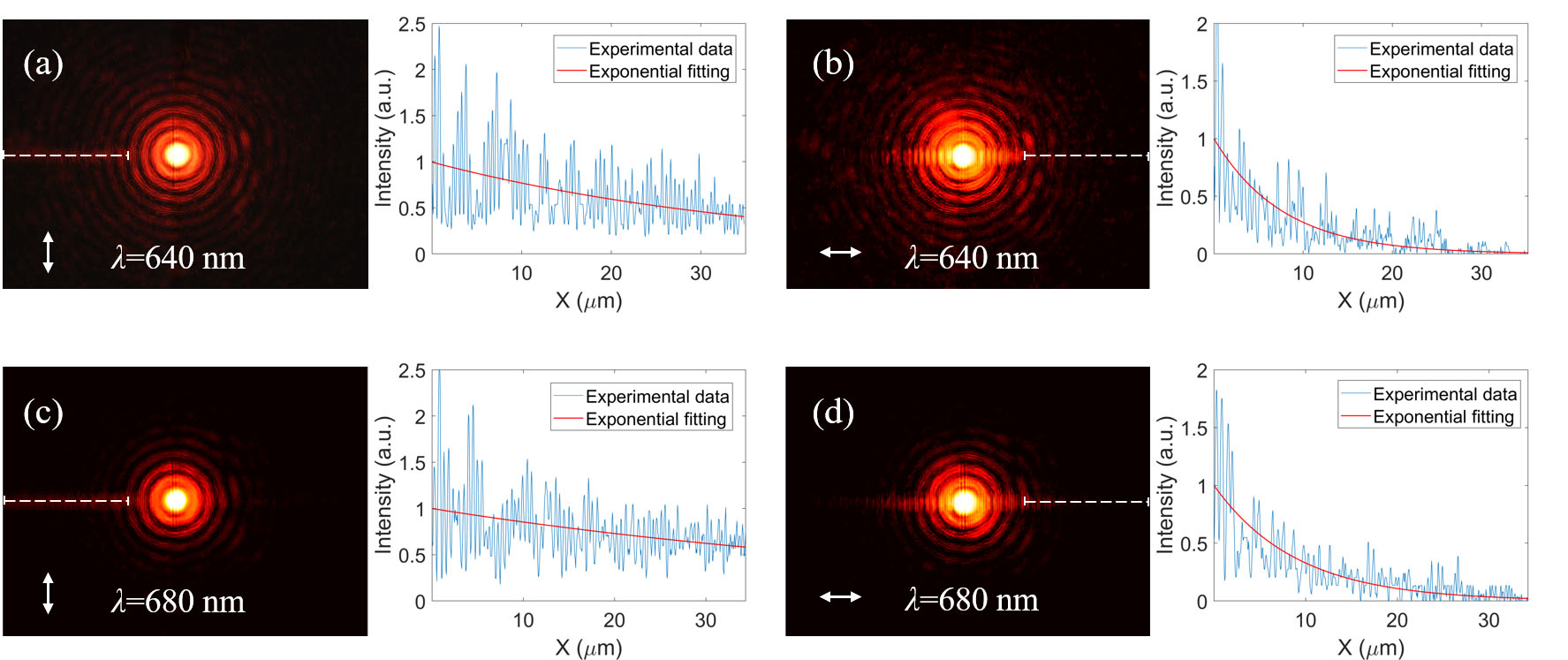


**Supplementary Figure S5.** FFP images for TE and TM BSWs at 640 nm and 680 nm respectively. The blue lines on the right panels are cross-sectional plots along the white dashed lines with red lines the corresponding exponential fittings. The propagating length can be estimated as 38.7 μm (TE BSWs) and 7.7 μm (TM BSWs) at a wavelength of 640 nm from the exponential fittings in (a-b). While the propagating length can be estimated as 63.6 μm (TE BSWs) and 10.6 μm (TM BSWs) in (c-d).


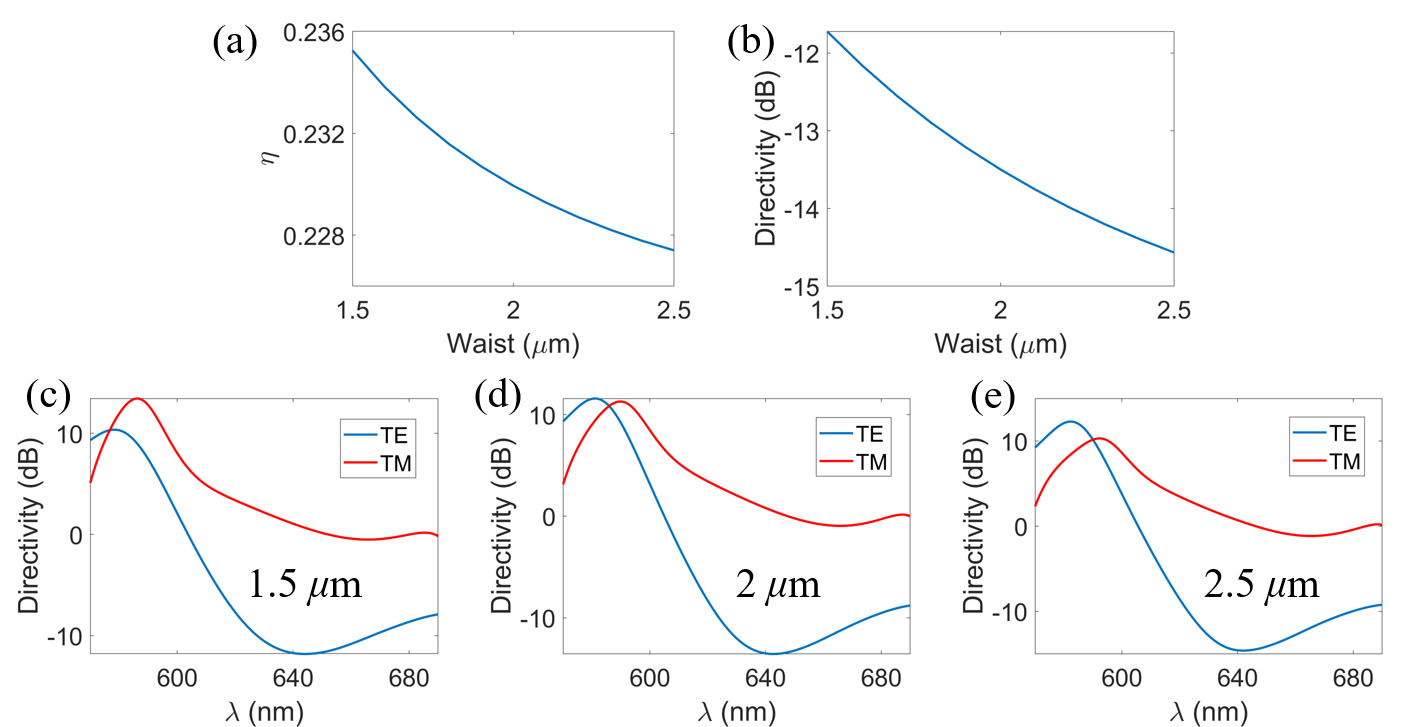


**Supplementary Figure S6.** (a-b) Simulated coupling efficiency and directionality variation on the waist under the illumination of TE polarized Gaussian beam with a wavelength of 640 nm. (c-e) Wavelength dependence of the directivity for TE (blue solid line) and TM (red solid line) polarized BSWs for a waist of (c) 1.5 *μ*m (d) 2 *μ*m (e) 2.5 μm. From (c-e), the spectral and polarimetric routing behavior is rarely influenced by beam waist.


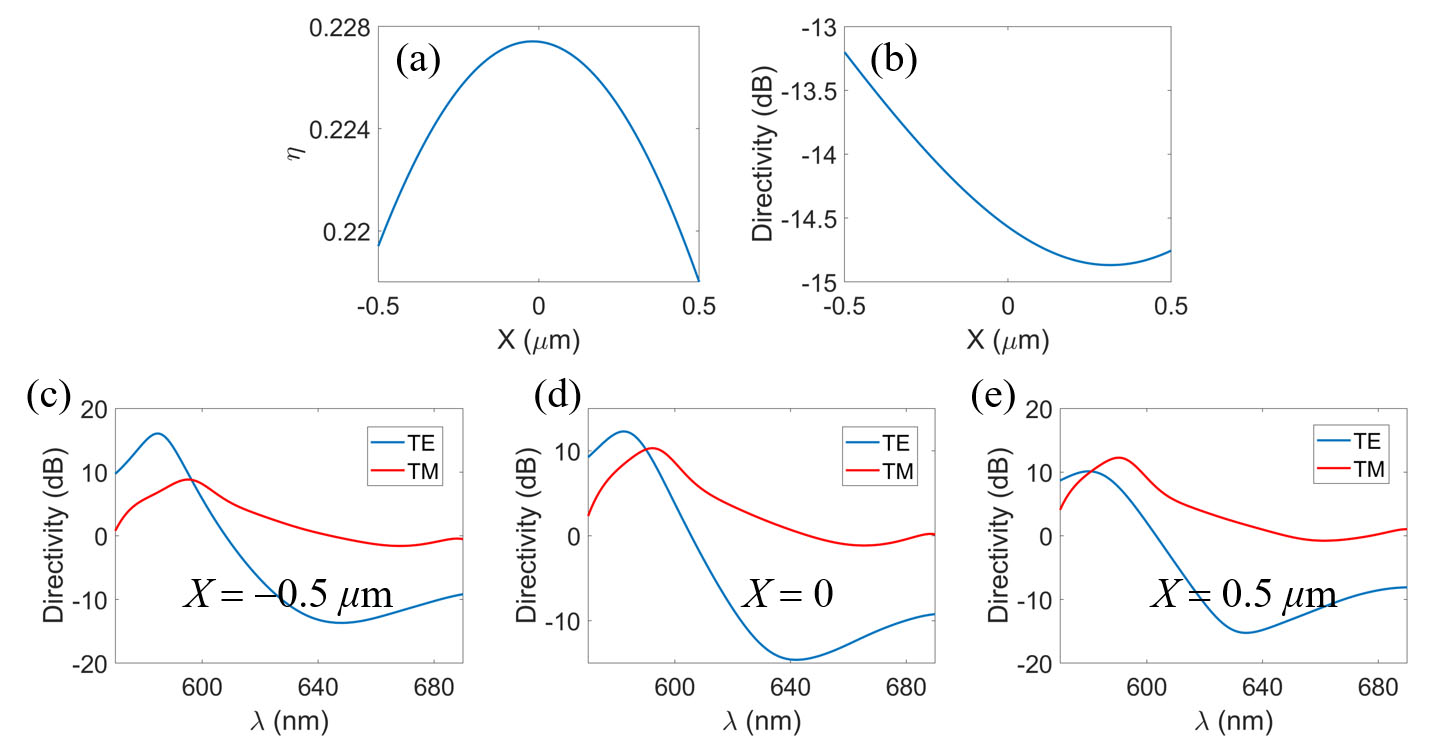


**Supplementary Figure S7.** (a-b) Simulated coupling efficiency and directionality variation on the beam position under the illumination of TE polarized Gaussian beam with a wavelength of 640 nm and waist of 2.5 *μ*m. (c-e) Wavelength dependence of the directivity for TE (blue solid line) and TM (red solid line) polarized BSWs with beam position of (c) -0.5 *μ*m (d) 0 *μ*m (e) 0.5 *μ*m. From (c-e), the spectral and polarimetric routing behavior is rarely influenced by beam position.


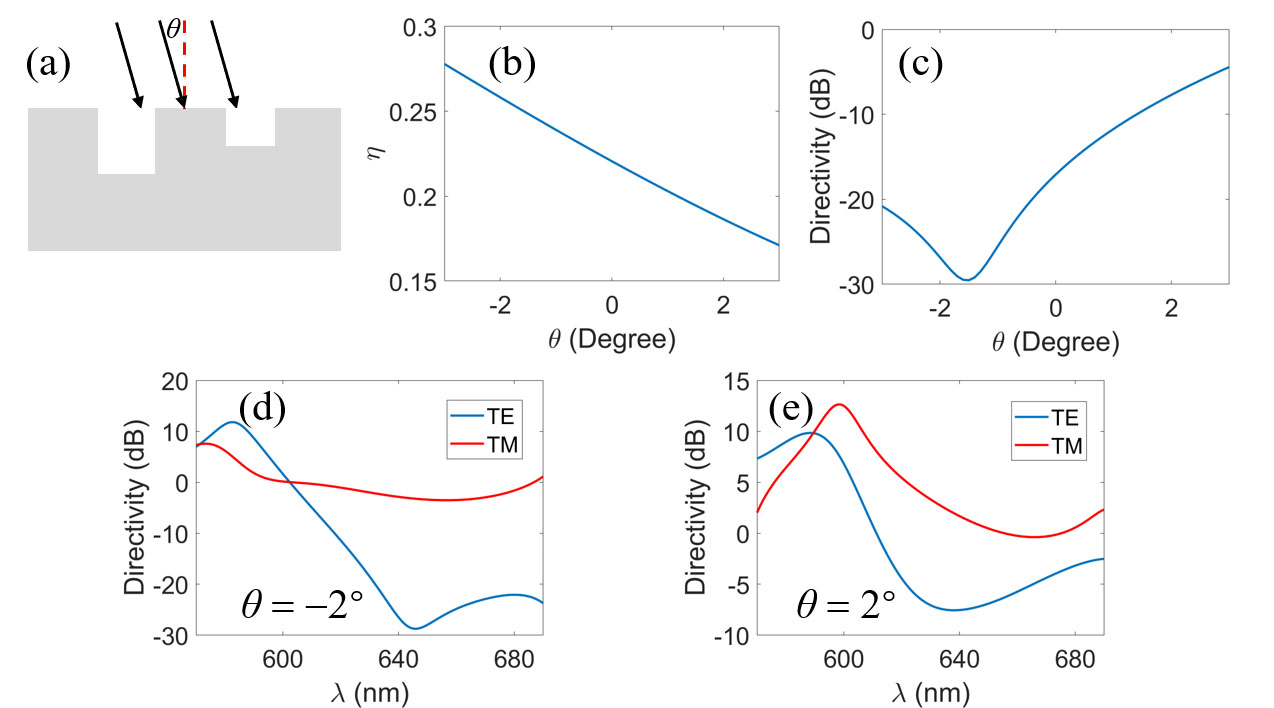


**Supplementary Figure S8.** (a) Schematics of light oblique incident on the structure. (b-c) Simulated coupling efficiency and directionality variation on the tilted angle the illumination of TE polarized Gaussian beam with a wavelength of 640 nm and waist of 2.5 *μ*m. (d-e) Wavelength dependence of the directivity for TE (blue solid line) and TM (red solid line) polarized BSWs with angle of (d) -2° (e) 2°. In (d), the directivity can reach near −30 dB for TE BSWs while the directional coupling is not clear for TM BSWs. In (e), the directivity for TM BSWs will be improved, while the directional coupling will be slightly suppressed for TE BSWs.
